# Assessing the 2004–2018 Fentanyl Misusing Issues Reported to an International Range of Adverse Reporting Systems

**DOI:** 10.3389/fphar.2019.00046

**Published:** 2019-02-01

**Authors:** Fabrizio Schifano, Stefania Chiappini, John Martin Corkery, Amira Guirguis

**Affiliations:** Psychopharmacology, Drug Misuse and Novel Psychoactive Substances Research Unit, School of Life and Medical Sciences, University of Hertfordshire, Hatfield, United Kingdom

**Keywords:** opioids, fentanyl, prescription drug misuse, opioid-related deaths, new psychoactive substances

## Abstract

**Objective:** A recent, global, increase in the use of opioids including the prescribing, highly potent, fentanyl has been recorded. Due its current popularity and the potential lethal consequences of its intake, we aimed here at analyzing the fentanyl misuse, abuse, dependence and withdrawal-related adverse drug reactions (ADRs) identified within the European Medicines Agency (EMA), the United Kingdom Yellow Card Scheme (YCS), and the United States Food and Drug Administration (FDA) Adverse Event Reporting System (FAERS) databases.

**Methods:** Descriptive analysis of both ADRs and related cases.

**Results:** The analysis of fentanyl-related misuse, abuse, dependence and withdrawal cases reported during years 2004–2018 to the EMA, the YCS, and the FAERS showed increasing levels overtime, specifically, EMA-related data presented two peaks (e.g., in 2008 and 2015), whilst the FAERS dataset was characterized by a dramatic increase of the ADRs collected over the last 18 months, and particularly from 2016. Some 127,313 ADRs (referring to *n* = 6,161 patients/single cases) related to fentanyl’s misuse/abuse/dependence/withdrawal issues were reported to EMA, with 14,287 being judged by the reporter as “suspect.” The most represented ADRs were: “drug dependence “(76.87%), “intentional product misuse” (13.06%), and “drug abuse” (7.45%). Most cases involved adult males and the concomitant use of other prescribing/illicit drugs. A range of idiosyncratic (i.e., ingestion/injection of transdermal patches’ fentanyl) and very high-dosage intake cases were here identified. Significant numbers of cases required either a prolonged hospitalization (192/559 = 34.35%) or resulted in death (185/559 = 33.09%). Within the same time frame, YCS collected some 3,566 misuse/abuse/dependence/withdrawal ADRs, corresponding to 1,165 single patients/cases, with those most frequently reported being “withdrawal,” “intentional product misuse,” and “overdose” ADRs. Finally, FAERS identified a total of 19,145 misuse/abuse/dependence/withdrawal-related cases, being “overdose,” withdrawal, and “drug use disorder/drug abuse/drug diversion” the most represented ADRs (respectively, 43.11, 20.80, and 20.29%).

**Conclusion:** Fentanyl abuse may be considered a public health issue with significant implications for clinical practice. Spontaneous pharmacovigilance reporting systems should be considered for mapping new trends of drug abuse.

## Introduction

### The Current “Opioid Crisis”

During recent years, a massive, worldwide increase (United Nations Office on Drugs, and Crime [UNODC], 2018a) in the prescription of opioids for pain has been recorded (Guevremont et al., 2018). This has been associated with increasing risks of diversion, abuse, morbidity and mortality, with a rising number of deaths and treatment admissions observed. Such “opioid crisis” (Throckmorton et al., 2018) started in 2013 (National Institute on Drug Abuse [NIDA], 2017; O’Donnell et al., 2017), and in recent years has reached the magnitude level of a public health issue, being tramadol, fentanyl and oxycodone the most involved molecules (Stannard, 2012; Centers for Disease Control and Prevention [CDC], 2014; Van Amsterdam and Van den Brink, 2015; Helmerhorst et al., 2017; Jordan et al., 2017; Floyd and Warren, 2018). In the United States in 2016, nearly 4% of the population aged 12 years and older reported a non-medical, past-year, use of prescription opioids. Compared with heroin use, which has been increasing each year since 2007, the non-medical use of prescription opioids has shown a stable trend in the past 5 years. Even though the most commonly misused prescription opioids reported in the National Survey on Drug Use and Health in the United States are hydrocodone, oxycodone, codeine and tramadol, fentanyl appeared to be on the rise (World Drug Report, 2018). According to the European Monitoring Centre for Drug and Drug Addiction [EMCDDA] (2018a), in addition to heroin, other opioid products have been seized in European countries, including tramadol, buprenorphine, methadone, but also fentanyl derivatives, with figures respectively, being: 3,553, 3,523, 1,245, and 738 seizures, with overtime increasing levels of availability of the latter. In Europe, fentanyl issues seem to be particularly relevant in Estonia (European Monitoring Centre for Drug and Drug Addiction [EMCDDA], 2018a). Considering data from the United States Unintentional Drug Overdose Reporting System (SUDORS), fentanyl has been detected in 56.3% of 5,152 opioid-related deaths during the months July–December 2016 (O’Donnell et al., 2017). In 2016, the United States synthetic opioid-related deaths accounted for 30.5% of all drug overdose fatalities and 45.9% of all opioid-related deaths, with a 100% increase in the rate of these fatalities compared with the previous year (Center for Disease Control and Prevention [CDC], 2018a,b; Seth et al., 2018; World Drug Report, 2018; News Release, 2018).

Although Europe does not seem to face a problem of the same scale of the United States (Van Amsterdam and Van den Brink, 2015), after a downward trend in opiate use since the late 1990s and until 2013, opiate use rates and drug-related deaths have started increasing again in Western and Central Europe (United Nations Office on Drugs, and Crime [UNODC], 2018b), In 2016, the use of opioids (e.g., heroin, but also: methadone, buprenorphine, fentanyl, codeine, morphine, tramadol and oxycodone) was reported as the main reason by 37% of all clients who entered European specialized drug clinics (European Monitoring Centre for Drug and Drug Addiction [EMCDDA], 2018a). The United Kingdom, Spain, and Sweden present with the most significant levels of non-medical, opioid-based, prescription drug use (United Nations Office on Drugs, and Crime [UNODC], 2018b). In France, the national OPPIDUM (“Observation of illegal drugs and misuse of psychotropic medications”) program of the French addictovigilance network (Frauger et al., 2017), anonymously collects information on drug abuse and dependence observed in patients recruited in specialized drug care centers. In 2015, OPPIDUM reported high percentages (77%) of opiate maintenance treatment among a number of 5,003 drug users, highlighting the emerging misuse of a range of synthetic opioids, such as tramadol, oxycodone, and fentanyl. In Germany, during years 2005–2014, a number of 242 fentanyl-related overdose fatalities were reported, with the onset of fentanyl-related deaths following the local launch of transdermal fentanyl matrix patches in 2004 (Sinicina et al., 2017).

### The Emerging Threat of Illicit Fentanyl Products

In Europe, during 2016–2017 fentanyl has been involved in more than 250 fatalities (European Monitoring Centre for Drug and Drug Addiction [EMCDDA], 2018a); this may have been associated with fentanyl derivatives’ high potency, possible use by opioid-naive individuals, and recently increased drug availability levels. In the United Kingdom, in early 2017, fentanyl and its synthetic analogs, such as carfentanil, butyryl fentanyl, fluorobutyrylfentanyl, furanylfentanyl, and alfentanil, have been detected in 25 drug-related fatalities (Hikin et al., 2018).

Different fentanyl derivatives have been developed by the legitimate pharmaceutical industry by adding various substituents to the basic molecule in order to modify the potency (European Monitoring Centre for Drug and Drug Addiction [EMCDDA], 2015). The same approach has been mimicked by chemists in clandestine laboratories to produce new, illicit, fentanyl derivatives. In fact, an overall number of 38 new synthetic opioids have been detected in the European drug market since 2009, out of these, 28 pertained to the fentanyls’ category (European Monitoring Centre for Drug and Drug Addiction [EMCDDA], 2018a). The vast range of illicit fentanyls (Gladden et al., 2016; National Institute on Drug Abuse [NIDA], 2017; Armenian et al., 2018; Center for Disease Control and Prevention [CDC], 2018b; Pichini et al., 2018) are manufactured in a range of non-EU countries (Macmadua et al., 2017; European Monitoring Centre for Drug and Drug Addiction [EMCDDA], 2018b) and then made available from both the streets and the web to be typically self-administered either on their own or in combination with remaining psychoactives. Users are often unaware of the contents of the substance they are taking, which inevitably leads to a great number of fatal overdoses (Helander et al., 2017; Zawilska, 2017; Kuczyñska et al., 2018; World Drug Report, 2018).

### Fentanyl, Clinical Pharmacological Issues

Fentanyl is an extremely fast-acting synthetic narcotic analgesic, first approved as an anesthetic in 1963 (Drummer, 2018; Food and Drug Administration [FDA], 2018). Currently it is available for intravenous (I.V.) and intramuscular (I.M.) injection, but also as transdermal patches, quick acting lozenges, and dissolving tablets and films. Fentanyl has a potency of at least 80 times that of morphine, and it is indicated for the treatment/management of chronic, malignant, and post-surgical pain conditions (Stanley, 2014; Drummer, 2018). Fentanyl is a narcotic analgesic acting predominately at the μ-opiate receptor (Drummer, 2018). Apart from the analgesic characteristics, the fentanyls as a group produce drowsiness, relaxation and euphoria, the latter being less pronounced than with heroin and morphine. The most common side effects include nausea, dizziness, vomiting, fatigue, headache, constipation, anemia, and peripheral oedema (European Monitoring Centre for Drug and Drug Addiction [EMCDDA], 2015; Prekupec et al., 2017). A range of severe toxicity effects, including muscle rigidity, seizures, overdoses, and death due to respiratory arrest, have been reported as well (Zawilska, 2017). Tolerance and dependence develop rapidly after repeated use. Characteristic withdrawal symptoms (sweating, anxiety, diarrhea, bone pain, abdominal cramps, shivers or “goose flesh”) occur when use is stopped too quickly (Zawilska, 2017). Serious interactions can occur when fentanyls are mixed with heroin, cocaine, alcohol and other CNS depressants, e.g., benzodiazepines. Sudden fatalities may be related to a cardiac arrest or severe anaphylactic reactions. The estimated lethal dose of fentanyl in humans is 2 mg. The recommended serum concentration for analgesia is 1–2 ng/ml and for anesthesia it is 10–20 ng/ml. Blood concentrations of approximately 7 ng/ml or greater have been associated with fatalities where poly- substance use was involved (European Monitoring Centre for Drug and Drug Addiction [EMCDDA], 2015). Whilst fatalities have been reported after therapeutic use, many deaths have occurred as a result of the misuse of pharmaceutical products. Both used and unused fentanyl patches have been injected, smoked, snorted or taken orally with fatal consequences (Lilleng et al., 2004; Woodall et al., 2008; European Monitoring Centre for Drug and Drug Addiction [EMCDDA], 2015).

### Fentanyl and Derivatives’ Misuse/Abuse Issues

The diversion of prescription fentanyl may involve individuals obtaining medication inappropriately through their profession, patients using their own prescribed fentanyl recreationally for a non-medically intended purpose, and subjects using a medication being prescribed to another person. Typical sources of medications included friends, family members, and online pharmacies (Novak et al., 2016).

Recreational fentanyl (also known by the street names “China White,” “Synthetic Heroin,” “Tango and Cash,” etc., Zawilska, 2017) consumption seems to be often associated with the use of other drugs such as heroin, other opiate/opioid medicines, alcohol, cocaine, benzodiazepines, psychostimulants, and antidepressants, and may lead to fatal and non-fatal overdoses (Uusküla et al., 2015; National Institute on Drug Abuse [NIDA], 2016; Alcohol and Drug Foundation [ADF], 2018; Armenian et al., 2018; Drummer, 2018; Kuczyñska et al., 2018).

Even under medical surveillance, the risk of overdose when injecting fentanyl would be significantly higher than when injecting heroin (Frisoni et al., 2018). Fentanyl overdoses may start suddenly, with a potentially lethal respiratory depression possibly being reached within 2 min as opposed to some 20–30 min after heroin use (Abdulrahim et al., 2018); moreover, the high rate of fentanyl deaths may be explained as well by the molecule polydrug consumption. Naloxone, often in repeated doses, followed by a significant amount of post-emergency clinical observation time (Greene et al., 2018; Santos et al., 2019) is used to treat fentanyl’s overdoses (Prekupec et al., 2017; Zawilska, 2017; Armenian et al., 2018; European Monitoring Centre for Drug and Drug Addiction [EMCDDA], 2018a), although at times this proves unsuccessful (Kuczyñska et al., 2018). A range of harm-reduction strategies have been implemented both in Europe (European Monitoring Centre for Drug and Drug Addiction [EMCDDA], 2016) and in the United States (New York City Health, 2017).

The improper use of transdermal patches, either by applying multiple patches on the body, or injecting/insufflating/inhaling (after volatilization) the contents of a discarded patch has been reported (National Institute on Drug Abuse [NIDA], 2017; Sinicina et al., 2017; Drummer, 2018; Jones et al., 2018; Kuczyñska et al., 2018). The fentanyl’s rewarding effects are increased when the drug is injected or self-administered with nasal sprays/e-liquids, which are vaped using electronic cigarettes (European Monitoring Centre for Drug and Drug Addiction [EMCDDA], 2018a).

Due to the growing fentanyl toxicity issues and their high abuse liability/dependence potential, all fentanyls approved for medical use are internationally controlled as Schedule II drugs under the Controlled Substance Act (National Institute on Drug Abuse [NIDA], 2018) and as a Class A drug under the Misuse of Drugs Act in the United Kingdom (GOV.UK, 1971). In 2017, also the fentanyl precursors 4-anilino-N-phenethylpiperidine (ANPP) and N-phenethyl-4-piperidone (NPP), have been added to Table 1 of the 1988 United Nation Convention, and in 2018 have been included under the European drug monitoring regulations (Abdulrahim et al., 2018; European Commission (EC), 2018). Nonetheless, over the last few years there have been growing concerns regarding a range of illicitly manufactured fentanyl analogs being used as new/novel psychoactive substances (NPS) (European Monitoring Centre for Drug and Drug Addiction [EMCDDA], 2015, 2018a,b; National Institute on Drug Abuse [NIDA], 2017; Pichini et al., 2018; Ventura et al., 2018). Although their chemistry is similar to fentanyl, they are not routinely detected (O’Donnell et al., 2018) and may present with a higher/much higher potency than the parental compound. Most popular fentanyl derivatives include: carfentanil (approximately 10,000 times more potent than morphine), acetyl-fentanyl (about 15 times more potent than morphine), and butyrfentanyl (30 times more potent than morphine; Prekupec et al., 2017; Zawilska, 2017; Pichini et al., 2018).

**Table 1:** Data relating to fentanyl misuse/abuse/dependence/withdrawal-related ADRs reported to the European Medicines Agency (EMA), the Medicines and Healthcare products Regulatory Agency (MHRA), and the Food and Drug Administration (FDA) pharmacovigilance databases, 2004–2018.

Characteristics	EMA EV Data	MHRA YCS Data	FAERS Data
Fentanyl misuse/abuse/ dependence/withdrawal -related issues	127,313 (e.g., 6,161 Individual cases); 14,287 “suspect” (e.g., 559 CASES)	3,566 Reactions (e.g., 1,165 Individual cases)	19,145 Individual cases
Most frequently reported ADRS’ issues	Drug dependence (76.9%), Intentional product misuse (13.1%), Drug abuse (7.5%)	Withdrawal (24.9%), Intentional product misuse and use issues (19.6%), Overdose (17.6%)	Overdose (42.1%), Withdrawal (20.5%), Drug abuse (20.0%)
Age (years)	Adult	Adult	N/A
	Age group 35–64 (229/559 = 41%)	Age group:	
		50–59 (164/1,165 = 14.1 %)	
		40–49 (144/1,165 = 12.4%)	
		60–69 (141/1,165 = 12.1 %)	
Gender	Male (M/F: 319/209 = 1.52)	F (M/F: 434/657 = 0.66)	N/A
Fentanyl as sole drug or in combination	Fentanyl sole drug: 307/559 = 54.9% cases.	N/A	N/A
	Concomitant drugs reported: other opioids (69.0%), cocaine (9.5%), benzodiazepines (6.8%), cannabis (5.6%), and ethanol (5.2%)		


#### Aims

To assess fentanyl misuse/abuse/dependence and withdrawal-related issues, we aimed here at analyzing the European Medicines Agency [EMA] EudraVigilance (EV) database, and comparing it with the United Kingdom Medicine and Healthcare products Regulatory Agency (MHRA) Yellow Card scheme (YCS), and the United States Food and Drug Administration [FDA] Adverse Event Reporting System [FAERS] databases.

## Materials and Methods

### EudraVigilance (EV) Features

European Medicines Agency (EMA) data were collected through EV, i.e., the pharmacovigilance dataset which manages and analyses information on suspected adverse reactions to medicines which have been authorized in the European Economic Area (EEA) (European Medicines Agency [EMA], 2007). This dataset is a centralized database of all suspected adverse drug reactions (ADRs) submitted to EMA through Individual Case Safety Reports (ICSR), providing information related to an individual case of a suspected side effect due to a medicine. Specifically, an ADR is defined as “…a response which is noxious and unintended, and which occurs at doses normally used in humans …f An ADR, contrary to an adverse event, is characterized by the suspicion of “…a causal relationship between the drug and the occurrence…” (European Medicines Agency [EMA], 2017). The ADRs here considered were, *per se*, spontaneous and unsolicited communications reported by both Regulatory Authorities of the EU Member States where the reaction occurred, and/or by the Marketing Authorization Holders for those ADRs occurring outside the EEA. Consistent with the EV Access Policy (European Medicines Agency [EMA], 2016), data were made available here after a formal, *ad hoc*, request regarding fentanyl, including the following molecules: “fentanyl,” “fentanyl hydrochloride,” and “fentanyl buccal,” with all pharmaceutical combinations having been excluded. For each reported case, EV recorded Level 2A information, meaning: general information on the ADR (e.g., code number of the ADR, sender type, sender organization, type of report, date when the report was first received, primary source country, reporter qualification, seriousness of the case, and medical confirmation of the case), information on the patient (age, sex, weight, and height), type of reaction/event, drug information (e.g., type of drug, dosages, administration route, and duration), including concomitant licit and illicit drugs, medical history and comments, outcome of the reaction including death, literature references (European Medicines Agency [EMA], 2016, 2018). Each ADR was recorded according to the Medical Dictionary for Regulatory Activities (MedDRA) (Medical Dictionary for Regulatory Activities [MedDRA], 2017), and listed through Preferred Terms (PT). PTs are defined as “distinct descriptor (single medical concept) for a symptom, sign, disease diagnosis, therapeutic indication, investigation, surgical or medical procedure, and medical social or family history characteristic” (Medical Dictionary for Regulatory Activities [MedDRA], 2017).

In the data analysis of our study we included the ADR identified by the following PT: “dependence,” “drug abuse,” “drug abuser,” “drug dependence,” “drug diversion,” “drug withdrawal syndrome,” “intentional product misuse,” “intentional product use issue,” “intentional overdose,” “overdose,” “substance use,” “substance abuse,” and “withdrawal syndrome” (Medical Dictionary for Regulatory Activities [MedDRA], 2017). The following ADRs were excluded from the analysis: “accidental exposure,” “accidental overdose,” “drug administration error,” “drug prescribing error,” “toxicity to various agents,” “medication error,” and “off-label use.” ADRs’ numbers differed from those referring to single patients, since different reporters/senders could have independently flagged the same ADR to EMA, or several ADRs (involving various organ classes and so identified with specific PT) related to the primary searched ADRs (abuse/misuse/dependence and withdrawal ADRs) for the same patient could have been reported as well. A descriptive analysis of ADRs and cases (which were unequivocally identified by an EV local number) was then performed, according to the information provided (Medical Dictionary for Regulatory Activities [MedDRA], 2017).

### Access to the UK MHRA YCS and FAERS Pharmacovigilance Datasets

In order to obtain a better understanding of prescribing fentanyl misuse issues, publicly accessible, 2004–2018, data from both the UK MHRA YCS (Medicines and Healthcare products Regulatory Agency [MHRA], 2018b) and the FAERS (Food and Drug Administration [FDA] Adverse Event Reporting System [FAERS], 2018) were here analyzed as well. These data are made available via online public dashboards.

The YCS collects information on a range of ADRs spontaneously reported from healthcare professionals, members of the public, and pharmaceutical companies, this information are then entered onto the MHRA’s ADR database by a team of safety experts to assess the likelihood of causal relationship between the drug and the reported reactions. The YCS publishes cumulative listings of all suspected ADRs received through interactive Drug Analysis Profiles (iDAPs) (Medicines and Healthcare products Regulatory Agency [MHRA], 2018a). After selecting the iDAP related to “fentanyl,” a general overview of data relating to: age, gender, and type of reactions (organized by System Organ Class-SOC and MedDRA Preferred Terms – PTs) was made available online. A range of filters to the database were then applied here, with the time-frame and reactions selected being those used for the EV dataset (Medical Dictionary for Regulatory Activities [MedDRA], 2017).

The FAERS is a database that contains a range of voluntarily submitted adverse event reports (Food and Drug Administration [FDA] Adverse Event Reporting System [FAERS], 2018), with these events being coded using terms from the MedDRA dictionary (Medical Dictionary for Regulatory Activities [MedDRA], 2017). Searching for “fentanyl,” “fentanyl hydrochloride,” and “fentanyl buccal,” we gained access to a range of FAERS-related data, which were then properly filtered according to the type of reaction, consistent with the above described EMA and YCS data extraction modalities.

#### Ethics Statement

Complying with the European Data Protection legislation (e.g., Regulation (EC) No 45/2001EMA, European Medicines Agency [EMA] (2016)), the protection of privacy and integrity of individuals is guaranteed. Thus, all EMA data are fully and completely de-identified/anonymized; therefore, any patient identifier is not being disclosed. Similarly, both the YCS data and FAERS data are completely anonymized and fully de-identified. The study has been approved by the University of Hertfordshire Ethics’ Committee (reference number LMS/PGR/UH/03234, March 5, 2018).

## Results

**FIGURE 1:**
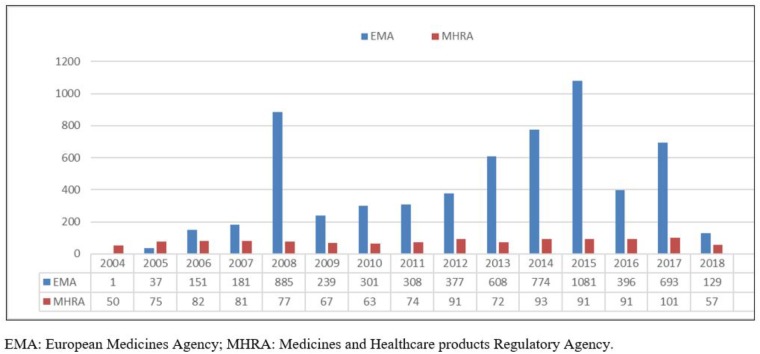
2004–2018 EMA and MHRA misuse/abuse/dependence/withdrawal-related fentanyl cases. EMA, European Medicines Agency; MHRA, Medicines and Healthcare products Regulatory Agency.

The analysis of the two ADR-related datasets showed an overall increase in the number of reports since 2004 to 2018, with two peaks in the trend having been identified in the EV database, respectively, in 2008 (885 ADRs) and 2015 (1,081 ADRs). Conversely, YCS data remained broadly stable at relatively low levels, being slightly increasing from 2014, with 101 cases reported in 2017 (Figure 1).

### Analysis of Data From the EV Database

During the period 2004–2018, the EMA EV system received a total of 127,313 ADRs (referring to *n* = 6,161 patients/single cases) relating to fentanyl misuse/abuse/dependence/withdrawal issues (Table 1). Out of these 127,313 ADRs, some 14,287 (corresponding to 559 patients) were identified following a further filtering exercise, whilst considering: (a) the PTs selected, and (b) the “suspect” fentanyl role in causing the index ADR case. The most represented ADRs were: “drug dependence” (10,982/14,287 ADRs, 76.87% of the total), “intentional product misuse” (1,866 ADRs, 13.06% of the total), and “drug abuse” (1,065 ADRs, 7.45%). Most reports (17.2%) were related to ADRs occurred in the United States and were posted by clinicians (10.82%). Out of these 559 individual cases, 429 (76.74%) were males (M/F: 319/209 = 1.52) in the 35–64 years-old age range; conversely, 10 subjects were younger than 12, and all had been diagnosed with an iatrogenic opioid withdrawal syndrome. Significant levels of ADR cases required either a prolonged hospitalization (192/559 = 34.35%) or resulted in death (185/559 = 33.09%).

Among the 185 fatal cases, the most reported causes of death were: toxicity to various agents (26/185 = 14.05%), drug abuse (19/185 = 10.27%), and overdose (18/185 = 9.73%). Although co-morbidity data went here typically unreported, chronic pain conditions (*n* = 36) were the most frequently mentioned medical conditions, whilst most typical psychiatric diagnoses included mood (55 cases) and anxiety (33 cases) disorders.

Although in most cases fentanyl was identified on its own (307/559 = 54.9% cases) concomitant drugs most typically mentioned in the EMA database included: remaining opiates/opioids (174/252 = 69%), cocaine (24/252 = 9.5%), benzodiazepines (17/252 = 6.8%), and cannabis (14/252 = 5.6%). Even though fentanyl’s route of administration was infrequently reported (e.g., oral: 41/559 = 7.3%, and transdermal: 33/559 = 5.9%), a range of idiosyncratic ways of administration/high dosage intake were here described, e.g.: 23 cases of transdermal patches’ ingestion, 10 cases of fentanyl nasal administration/inhalation, and 10 cases of intravenous/parenteral use, with a case of tampered transdermal patches’ injection on some 30 times a day having been reported. In terms of idiosyncratic dosages, a patient was here reported to be self-administering with 800 mcg 2–3 times/day, whilst another was daily self-administering with 11.56 mg of fentanyl transdermal patch. Where available, most typically reported blood toxicology screening results were in the range of: up to 10 ng/ml (7/559 cases), 10–50 ng/ml (16/559 cases), and in excess of 100 ng/ml (2/559 cases, in 1 case this was 313 ng/ml).

### Analysis of the YCS and FAERS Databases

Analysis of the 2004–2018 fentanyl YCS iDAPs identified some 3,566 fentanyl misuse/abuse/dependence/withdrawal-related ADRs, corresponding to 1,165 single patients/cases (Table 1). ADR numbers showed an increase over time (Figure 1), with a peak in 2017 (101 reactions). Female subjects (F/M: 657/434 reports), aged 40–59 years (308 reports), were most typically involved. Out of all reactions, “withdrawal” (79 reactions), “intentional product misuse and use issues” (62 reactions), “overdoses” (56 reactions), and “addiction/dependence/drug dependence” (45 reactions) were the most typically represented ADRs (Table 1). Moreover, in the 2004–2018-time frame, the FAERS database identified a total (e.g., all causes ADRs) of 78,885 instances. After completion of the above-described filtering exercise, a total of 19,145 misuse/abuse/dependence cases/patients were here identified. Most frequently mentioned reports related to: “overdose” (*n* = 8,255/19,145, 43.11%), “withdrawal syndrome” (*n* = 3,983, 20.80%), “drug use disorder/drug abuse/drug diversion” (*n* = 3,886, 20.29%), “intentional product use issues/misuse” (*n* = 1,829, 9.55%), and “drug dependence” (*n* = 1,462, 7.64%) (Table 1).

## Discussion

This unprecedented, large scale, research study aimed at systematically identifying and analyzing a total of some 26,500 fentanyl misuse/abuse/dependence/withdrawal cases. Present data were extracted from a range of high-quality (Schifano and Chiappini, 2018) pharmacovigilance databases, such as the EV (providing description on a total of unfiltered 6,161 misuse/abuse/dependence/withdrawal cases), the United Kingdom YCS (1,165 cases), and the United States FAERS (19,145 cases). Indeed, these data seem to once again confirm that non-medical prescription high potency opioid use is a major public health concern both in Europe (European Monitoring Centre for Drug and Drug Addiction [EMCDDA], 2015), and in the United States (Ali et al., 2017; Mital et al., 2018). According to European Monitoring Centre for Drug and Drug Addiction [EMCDDA] (2015) data, the main consumers in the EU in 2008 per million inhabitants per day were Belgium (13,601 Defined Daily Doses or S-DDD), Germany (13,341 S-DDD), and Austria (10,143 S-DDD). Moreover, even though illegally diverted fentanyl is a relatively marginal phenomenon in most EU countries, in Estonia as many as 70% of applicants for treatment services in 2009 reported fentanyl as their primary drug. Consistent with this, Han et al. (2017) in carrying out the 2015 National Survey on Drug Use and Health (NSDUH) exercise, analyzed data from 51,200 subjects who completed an *ad hoc* survey interview. They estimated that out of 91.8 million (37.8%) United States non-institutionalized adults who used prescription opioids, 11.5 million misused them, and 1.9 million had a use disorder. Being relief of physical pain the most commonly reported motivation for misusing prescription opioids (Han et al., 2017), developing new pain treatments would possibly reduce the access to opioids in high-risk patients (National Institute on Drug Abuse [NIDA], 2017). A further challenge may be constituted by identifying new opioid formulations with physical or pharmacologic deterrents to reduce tampering (Stanos et al., 2012; Passik, 2014).

After many years of stability, fentanyl ADRs seemed to have peaked here over the last 10 years and in the United States even more dramatically just over the last 2 years or so. Although it is possible that high levels of reporting were facilitated by a recently growing awareness on fentanyl misuse, and may somehow mirror as well the increasing rates of worldwide availability of this medication (United Nations Office on Drugs and Crime [UNODC], 2017), present figures are in line with previous findings (Ali et al., 2017). However, increasing rates of fentanyl misuse/abuse issues may have been facilitated as well by the high number of pro drug websites/users’ fora, where proper advice on how to both tamper fentanyl patches and best enjoy the related intake experience as well are given (Drugs-forum, 2017; The hive, 2018).

Possibly because of its high potency (Frisoni et al., 2018), fentanyl prescribing was here reported in a number of cases to be associated with iatrogenic dependence/withdrawal issues. It is a further reason of concern, however, that fentanyl was here self-administered either in idiosyncratic ways (i.e., parenteral, ingesting the transdermal patches) or at high/very high dosages to achieve significant blood levels (Frisoni et al., 2018). A large proportion of EMA ADR cases (e.g., roughly two out of three) was here associated either with a prolonged hospitalization or resulted in death. Fatalities related to novel synthetic opioids and fentanyls should be investigated using a multidisciplinary approach, aiming at framing each case and directing the investigations toward targeted toxicological analyses. This approach should be adopted routinely (Hikin et al., 2018), and especially in cases of death from uncertain or questionable causes (Lucyk and Nelson, 2017; Prekupec et al., 2017; Drummer, 2018). New approaches for the detection of potential unknown psychoactive substances, e.g., in the Emergency Departments, need to be developed, in order to identify and classify compounds that are new to the market and absent from existing chemical libraries (Assi et al., 2015; Guirguis et al., 2017; Calvo-Castro et al., 2018; Food and Drug Administration [FDA], 2018; News Release, 2018).

Although in some 54.9% of EMA ADRs a fentanyl intake was reported on its own, a range of both prescribing (e.g., remaining opiates/opioids, benzodiazepines), and recreational (e.g., cocaine and cannabis) psychotropics was here identified as well. Whilst these combinations are likely to lead to intoxication or death (Haukka et al., 2018), they may reflect the characteristics of clients prescribed with fentanyl, e.g., frequently affected by chronic pain conditions, anxiety, and depression, at times presenting as well with a history of drug misuse (Hughes et al., 2016).

Intake of high fentanyl dosages was possibly associated here with the need to relieve pain, whilst attempting to cope with the molecule’s increasing levels of tolerance overtime (Han et al., 2017). Nonetheless, fentanyl recreational value (Frisoni et al., 2018) should not be overlooked (Drummer, 2018). High fentanyl dosages may be associated with respiratory arrest, pulmonary oedema, chest wall rigidity and apnoea. A reported uncommon intoxication symptom is chest pain, with non-specific T-wave changes on the electrocardiogram, mimicking an acute coronary syndrome (for a thorough review, see Frisoni et al., 2018).

## Limitations

Even though the study of spontaneous reporting systems, such as EV, the YCS, and the FAERS, should be considered as a starting point for mapping the new trends of abuse, including the abuse of prescription drugs, the analysis of voluntarily reported ADRs may have some limitations. These pharmacovigilance database approach limitations include likely underreporting, reporting bias, and lack of access to the full range of available data. Some ADRs may be signaled several times by different reporters, therefore the number of suspected ADRs can be different to the number of cases as one individual case may refer to several suspected ADRs. Moreover, a report may describe different information, sometimes lacking useful data, such as medical history, dosages and route of administration, or cause of death when the outcome is fatal. Case reports of suspected ADRs do not confirm that a certain effect in a patient has been caused by a specific medicine (European Medicines Agency [EMA], 2011; Food and Drug Administration [FDA] Adverse Event Reporting System [FAERS], 2018; Medicines and Healthcare products Regulatory Agency [MHRA], 2018b), but may be used for detecting and assessing eventual safety issues to be investigated. Again, the worldwide fentanyl prescribing rates were not available here and, due to fentanyl’s availability on the black and gray (semi-legal) markets, the true incidence of the misusing phenomenon may not be calculated.

Finally, because of only partial consistency of data collection of the datasets here examined, analyzing and comparing figures may prove problematic. Indeed, MHRA and FAERS public dashboards provided here only a portion of available data (e.g., excluding diagnoses, medical histories, fentanyl dosages and concomitant drugs ingested) relating to the cases reported.

## Conclusion

Fentanyl abuse may be considered a public health issue, with enormous implications for the clinical practice. A national registry of patients to monitor and check opioid prescribing to high-risk patients would be helpful, this may improve the patient safety levels, whilst providing more focused epidemiological data regarding prescribing patterns (Mordecai et al., 2018). With the aim of reducing the number of people addicted to opioids, the FDA strategy is now to pose a control on prescription duration and doses for patients (Floyd and Warren, 2018; Food and Drug Administration [FDA], 2018).

In terms of prevention of the opiate epidemic and harm reduction strategies, it is here suggested that users should play an active role in helping drafting overdose education and abuse-deterrent strategies. Prompt referral/self-referral to treatment programs has been reported as being an effective intervention (Suzuki and El-Haddad, 2017; Ralphs and Gray, 2018; Schiller and Mechanic, 2018; Younga et al., 2018). In Europe, a range of evidence-based prevention programs have been implemented over the last few years (European Monitoring Centre for Drug and Drug Addiction [EMCDDA], 2017). Supervised drug consumption facilities and “take-home” naloxone programs, making the medication available to opioid users and their partners/peers/families, alongside with training in overdose recognition and response, appear to be helpful in preventing deaths (European Monitoring Centre for Drug and Drug Addiction [EMCDDA], 2018a) and should be encouraged (Uusküla et al., 2015). Moreover, the Welsh Emerging Drugs and Identification of Novel Substances [WEDINOS], 2018, the Loop in the United Kingdom (Wearetheloop.org, 2018), the Drug Information and Monitoring System in the Netherlands (Drug Information and Monitoring System [DIMS], 2018 and the MANchester DRug Analysis and Knowledge Exchange [MANDRAKE], 2018) are some drug awareness/harm reduction European projects aiming at monitoring the illegal drug market, identifying public health threats at an early stage.

Physicians should be educated and invited to a responsible prescribing of drugs with a diversion potential, whilst carefully evaluating the possibility for some clients (e.g., people with a personal history of drug abuse) to be more vulnerable to drug misuse (Han et al., 2017).

## Author Contributions

FS conceived the conceptual idea of the manuscript and the proof outline. SC performed the literature review and the analysis of data from EMA and drafted the initial version of the manuscript. AG and JMC supervised the manuscript and contributed to the final version of the manuscript. FS approved the final content of the manuscript.

## Conflict of Interest Statement

FS is an Advisory Council on the Misuse Drugs (ACMD) member, United Kingdom, and an EMA Advisory board (psychiatry) member. The authors have no other relevant affiliations or financial involvement with any organization or entity with a financial interest in or financial conflict with the subject matter or materials discussed in the manuscript.
